# Loss and Gain of Tolerance to Pancreatic Glycoprotein 2 in Celiac Disease

**DOI:** 10.1371/journal.pone.0128104

**Published:** 2015-06-05

**Authors:** Martin W. Laass, Nadja Röber, Ursula Range, Lydia Noß, Dirk Roggenbuck, Karsten Conrad

**Affiliations:** 1 Children’s Hospital, Medical Faculty Carl Gustav Carus, Technische Universität Dresden, 01307 Dresden, Germany; 2 Institute of Immunology, Technische Universität Dresden, 01307 Dresden, Germany; 3 Institute for Medical Informatics and Biometry, Technische Universität Dresden, 01307 Dresden, Germany; 4 GA Generic Assays GmbH, 15827 Dahlewitz/Berlin, Germany; 5 Faculty of Science, Brandenburg University of Technology Cottbus-Senftenberg, 01968 Senftenberg, Germany; UFMG, BRAZIL

## Abstract

**Background:**

Autoantibodies against pancreatic secretory-granule membrane glycoprotein 2 (GP2) have been demonstrated in patients with Crohn’s disease but recently also with celiac disease (CD). Both entities are characterized by intestinal barrier impairment with increased gut permeability. Pathophysiological hallmark of CD is a permanent loss of tolerance to alimentary gliadin and a transient loss of tolerance to the autoantigen human tissue transglutaminase (tTG). Therefore, we explored the behavior of loss of tolerance to GP2 reported in CD.

**Methods:**

We assessed prevalences and levels of autoantibodies against GP2, CD-specific antibodies to endomysial antigens and tTG as well as Crohn’s disease-specific anti-*Saccharomyces cerevisiae* antibodies in sera of 174 patients with active CD, 84 patients under gluten-free diet (GFD) and 129 controls. Furthermore, we looked for an association between anti-GP2 antibody positivity and degree of mucosal damage in CD.

**Results:**

We found significantly elevated anti-GP2 IgA positivity in active CD patients (19.5%) compared to CD patients under GFD (0.0%) and controls (5.4%, p < 0.001, respectively). Anti-GP2 IgA levels correlated significantly with CD-specific antibodies (p < 0.001). Anti-GP2 autoantibody positivity disappeared under GFD similarly to CD-specific autoantibodies against tTG and endomysial antigens. For the first time, IgA antibody levels to GP2 are demonstrated to be associated with degree of villous atrophy according to Marsh classification.

**Conclusions:**

Anti-GP2 IgA seems to be associated with disease activity in a distinct subgroup of patients with CD. The observed loss of tolerance to GP2 in a subset of patients with CD is transient and disappears under GFD.

## Introduction

Pancreatic secretory granule membrane glycoprotein 2 (GP2) (OMIM 602977) has recently been discovered as the major autoantigenic target of Crohn’s disease-specific pancreatic autoantibodies [1–3]. GP2 was described first as major glycoprotein synthesized in the acinus cells of the exocrine pancreas, which is eventually released into the intestine along with zymogens, and described later as specific receptor on intestinal microfold (M) cells of the follicle-associated epithelium, additionally [4, 5]. GP2 modulates T-cell activation, proliferation, and apoptosis and is up-regulated on activated human T-cells [6]. It seems to down-regulate inflammatory and up-regulate regulatory cytokine secretion. GP2, similar to its renal homolog, the Tamm–Horsfall protein, is able to bind type-I fimbria, an adhesin expressed by *E*. *coli* and other Enterobacteria [7, 8]. Therefore, it is conceivable that GP2 secreted in pancreatic juice is part of innate immunity against bacterial contaminants in food and not involved in food digestion [9, 10]. Nevertheless, the physiological function of GP2, especially that of pancreatic GP2, is not yet fully understood [11].

Autoantibodies against GP2 found in about 30% of patients with Crohn's disease appear to be associated with distinct disease phenotypes of Crohn's disease: younger age, ileocolonic location, and stricturing behavior with perianal disease [12–18]. Recently, anti-GP2 antibodies have been detected in patients with active celiac disease (CD) and even refractory CD [19–21]. CD is an immune-mediated enteropathy caused by a specific response of intestinal T-cells to gluten and related prolamine proteins of wheat, rye, barley, and related cereals in genetically susceptible individuals. CD is characterized by a variable combination of gluten-dependent intestinal and extraintestinal clinical signs and symptoms, CD-specific antibodies such as endomysial antibodies (EmA) or autoantibodies against tissue transglutaminase (tTG), and the presence of villous atrophy in the small bowel [22]. The pathophysiological mechanisms resulting in loss of tolerance to gluten and tTG are still not fully understood.

Given the loss of tolerance to GP2 in CD and assuming a role of GP2 in antigen presentation and immunomodulation in the small intestine, we i) assessed the prevalence of autoantibodies against GP2 in active and inactive CD, ii) looked for an association between anti-GP2 antibody positivity and clinical phenotype, and iii) investigated anti-GP2 IgA and IgG in sera from patients with histologically proven CD at the time of diagnosis and under gluten-free diet (GFD).

## Materials and Methods

### Study population

We investigated sera from 174 pediatric and adult patients with active CD at the time of diagnosis. Furthermore, 84 CD patients under GFD were enrolled. Sera were collected at the university hospital Dresden between 1994 and 2013. Clinical data, including age at diagnosis, family history, and body mass index were recorded. Details of clinical and epidemiological data are summarized in Table 1. In 145 patients, diagnosis of CD was made before the age of 18 years. In all 29 patients suffering from type 1 diabetes mellitus, diagnosis of CD was made either at the same time or after diabetes was diagnosed.

**Table 1:** Clinical characteristics of the study groups.

	active CD	CD under GFD^a^	Controls
Number	174	84	129
Age at diagnosis (median, range)	7 (0–72)	4 (0–62)	14 (0–22)
Gender: female (%)	109 (62.6%)	59 (70.2%)	57 (44.2%)
Histology (Marsh 3)	153^b^ (87.9%)	-	-
Marsh 3a	28	-	-
Marsh 3b	60	-	-
Marsh 3c	65	-	-
Family history of CD ^c^	36 (20.7%)	8 (9.5%)	0 (0.0%)
Type 1 diabetes	24 (13.8%)	20 (23.8%)	0 (0.0%)

^a^ In this group, 51 patients of group 1 with active CD and follow-up serology under gluten-free diet (GFD) are included.

^b^ For the other 21 patients, the original histology was not available. In one patient diagnosis was done according to the new ESPGHAN guidelines without biopsy [18].

^c^ The 36 patients with family history of CD were from 23 different families.

celiac disease (CD), gluten-free diet (GFD).

The diagnosis of CD was made according to the revised criteria of the European Society of Pediatric Gastroenterology, Hepatology and Nutrition from 1990 and to the new guidelines from 2012 [22]. Patients with IgA-deficiency were excluded from the study.

*Controls*: As control groups, we included 68 apparently healthy children, who were admitted to our hospital for eye surgery correcting their strabismus, and 62 healthy medical students of our medical faculty. In one control individual, however, a formerly unknown CD was detected. She was excluded from further analysis. In the remaining control population there was no history and no clinical sign of any autoimmune disease.

### Antibody determination

Exocrine pancreatic autoantibodies and EmA were assessed in serum samples of patients and controls by indirect immunofluorescence (Euroimmun Medizinische Labordiagnostika AG, Lübeck, Germany).

IgA and IgG antibodies against GP2 were measured using commercially available ELISAs employing recombinant human GP2 isoform alpha (expressed in Spodoptera frugiperda 9 cells) from GA Generic Assays (Dahlewitz, Germany) according to the manufacturer’s instructions [16, 23]. A cut-off for positivity was set at 20 U/mL for both anti-GP2 IgA and IgG antibody evaluation as recommended by the manufacturer. Anti-tTG were detected by ELISA employing recombinant human tTG with a cut-off for positivity at 40 U/mL (GA Generic Assays) [24]. Values for anti-tTG >300 U/mL were set to 301 U/mL.

Anti-*Saccharomyces cerevisiae* antibodies (ASCA) (IgA and IgG) were detected by ELISA employing phosphopeptidomannan with a cut-off for positivity at 35 U/mL (GA Generic Assays) [25].

IgA-deficiency was excluded by nephelometric measurement of total IgA levels in serum. Aliquots of serum samples have been stored at −20°C until antibody assessment.

### Inhibition experiments

For inhibition experiments, one sample with high titer of anti-GP2 IgA and one sample with high titer of anti-tTG IgA were selected. Serum dilutions giving an optical density value of around 1.0 were pre-incubated with recombinant GP2 and tTG at decreasing concentrations (0–10 μg/mL) for 60 minutes at room temperature. After incubation, ELISA tests were performed as described above for each antigen concentration.

### Statistical analysis

Statistical analyses were performed using SPSS 21.0 (SPPS Inc. Illinois, USA). The Kolmogorov–Smirnov test was used to analyze the data for normality. The two-tailed, non-parametric Mann-Whitney and Kruskal-Wallis tests were used to test for statistically significant differences of independent samples in 2 or more groups, respectively. The non-parametric Wilcoxon test was employed to test paired samples. Comparison of prevalence rates between groups was performed by two-tailed Fisher's exact test. Correlations of antibody tests were assessed by Pearson or Spearman correlation coefficient. Values of p < 0.05 were considered significant.

### Ethics Statement

The study was approved by the ethics committee of the Faculty of Medicine of the Technical University Dresden (ethical permit number: EK 151052010). All patients and controls included in this study gave written informed consent. For children we obtained written informed consent from the next of kin, caretakers, or guardians respectively. The consent procedures and the informed consent documents, which were created specifically for this study, were approved by the ethics committee of the Faculty of Medicine of the Technical University Dresden (see ethical permit number above). The study was conducted in accordance with the principles of the Declaration of Helsinki (World Medical Association Declaration of Helsinki 1989).

## Results

### Prevalence and concentration of antibodies against GP2 in patients and controls

All patients with active CD had positive CD-specific autoantibodies with often very high levels (anti-tTG IgA >300 U/mL and EmA titer > 1:320) indicating severe villous atrophy (Table 2). Indeed, available histology data of patients with active CD (n = 153) demonstrated mucosal changes according to Marsh 3 classification. Patients with CD under GFD and in particular controls showed significantly lower prevalences of CD-specific autoantibodies (p < 0.001, respectively).

**Table 2:** Prevalence and levels of antibodies in patients and controls.

	active CD	CD under GFD	Controls	p-value
Antibody positivity				
EmA IgA (%)	172/173 (99.4)^a^	17/84 (20.2)	0/61 (0.0)	< 0.001^b^ ^,^ ^c^
Anti-tTG IgA (%)	166/174 (95.4)	11/72 (15.3)	0/129 (0.0)	< 0.001^b^ ^,^ ^c^
Anti-tTG IgG (%)	151/174 (86.8)	15/59 (25.4)	1/128 (0.8)	< 0.001^b^ ^,^ ^c^
Anti-tTG IgA/IgG (%)	173/174 (99.4)	15/59 (25.4)	1/128 (0.8)	< 0.001^b^ ^,^ ^c^
Anti-GP2 IgA (%)	34/174 (19.5)	0/84 (0.0)	7/129 (5.4)	< 0.001^b^ ^,^ ^c^
Anti-GP2 IgG (%)	8/174 (4.6)	3/82 (3.7)	2/126 (1.6)	1.000^b^, 0.201^c^
Anti-GP2 IgA/IgG (%)	35/174 (20.1)	3/82 (3.7)	9/126 (7.1)	< 0.001^b^, 0.002^c^
ASCA IgA (%)	9/160 (5.6)	0/33 (0.0)	1/68 (1.5)	0.362^b^, 0.288^c^
ASCA IgG (%)	22/167 (13.2)	0/33 (0.0)	0/68 (0.0)	0.029^b^, < 0.001^c^
ASCA IgA/IgG (%)	28/160 (17.5)	0/33 (0.0)	1/68 (1.5)	0.005^b^, < 0.001^c^
Antibody level (median, IQR)				
EmA (titer)	320.0 (160.0–640.0)	4.6 (2.8–8.8)	3.5 (1.6–7.2)	< 0.001^d^ ^,^ ^e^
Anti-tTG IgA (U/mL)	301.0 (174.6–301.0)	11.9 (4.3–25.9)	3.6 (2.6–4.7)	< 0.001^d^ ^,^ ^e^
Anti-tTG IgG (U/mL)	203.4 (64.2–301.0)	17.2 (5.9–41.9)	4.4 (3.1–6.5)	< 0.001^d^ ^,^ ^e^
Anti-GP2 IgA (U/mL)	4.6 (1.7–14.3)	1.6 (1.0–3.6)	1.7 (1.0–5.0)	< 0.001^d^ ^,^ ^e^
Anti-GP2 IgG (U/mL)	5.4 (3.5–8.9)	4.6 (2.8–8.8)	3.5 (1.6–7.2)	0.154^d^, < 0.001^e^
ASCA IgA (U/mL)	5.9 (3.3–9.9)	4.5 (2.8–9.0)	2.4 (1.9–3.7)	0.192^d^, < 0.001^e^
ASCA IgG (U/mL)	8.9 (3.9–16.6)	6.0 (4.0–11.0)	4.4 (3.0–8.7)	0.086^d^, < 0.001^e^

Prevalence of antibody positivity and antibody levels for endomysial autoantibodies (EmA), autoantibodies to tissue transglutaminase (anti-tTG) and glycoprotein 2 (anti-GP2) as well as anti-Saccharomyces cerevisiae antibodies (ASCA) in patients with active celiac disease (CD), CD under gluten-free diet (GFD) and 129 controls. Median antibody titers are given in U/mL (anti-GP2, anti-tTG, ASCA) or titer (EmA).

^a^ In one patient, EmA-IgA titre was 1:10 (borderline), but the other antibodies were positive and histology showed a Marsh 3c (total villous atrophy).

^b^ Fisher’s exact test, CD versus CD under GFD

^c^ Fisher’s exact test, CD versus controls

^d^ two-tailed, non-parametric Mann-Whitney test, CD versus CD under GFD

^e^ two-tailed, non-parametric Mann-Whitney test, CD versus controls

EmA endomysial antibodies, tTG tissue transglutaminase, GP2 glycoprotein 2, ASCA anti-Saccharomyces cerevisiae antibodies.

Anti-GP2 IgA was significantly more often positive in patients with active CD (19.5%) compared to controls (5.4%) and also to patients with CD under GFD (0.0%) (p < 0.001, respectively). In contrast, the prevalence of anti-GP2 IgG was not significantly higher in active CD patients compared with the other cohorts (p < 0.05, respectively). Like anti-GP2 IgA, ASCA IgG was more prevalent in patients with active CD compared with those under GFD and controls (p = 0.029, p < 0.001, respectively).

As expected, CD-specific autoantibody levels were all significantly higher in patients with active CD compared with those under GFD and controls (p < 0.001, respectively). Of the other antibodies investigated, only median values of anti-GP2 IgA and ASCA IgG in active CD patients were significantly elevated compared to those in patients under GFD and controls (Table 2).

We tested sera of 10 patients and two controls with the highest anti-GP2 IgA levels for autoantibodies against exocrine pancreas by indirect immunofluorescence. No reticulogranular or extracellular drop-like staining which is typical for exocrine pancreatic autoantibodies was seen.

### Correlation of anti-GP2 with CD-specific autoantibodies

**Fig 1:**
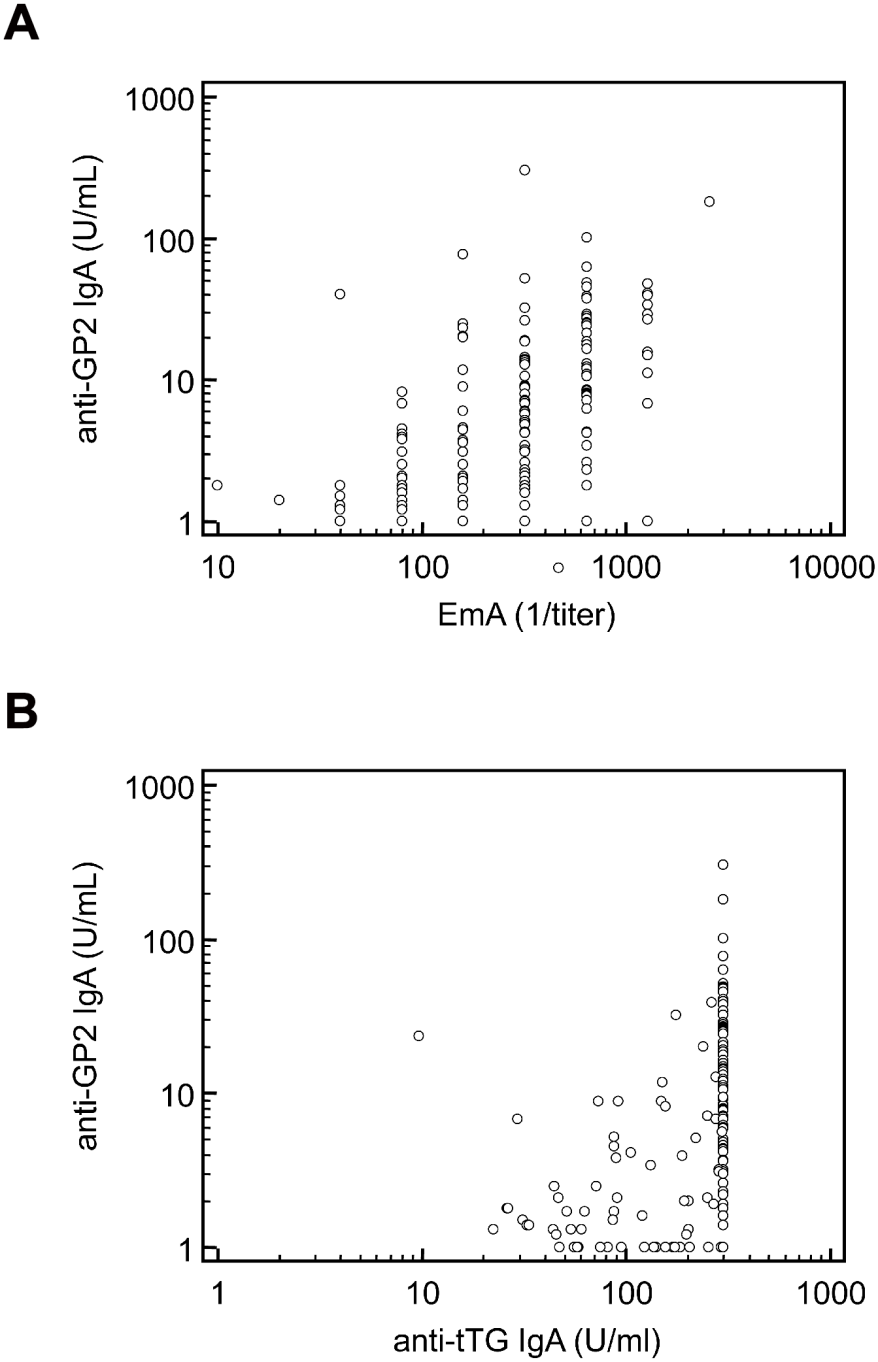
Correlation of anti-GP2 IgA with celiac disease (CD) specific antibodies. Association of anti-GP2 IgA with endomysial antibodies (EmA) (A) and anti- tissue transglutaminase (tTG) IgA (B) in 174 patients with active CD. (Spearman's coefficient of rank correlation for (A) 0.466 and (B) 0.494 (p<0.001, respectively).

A significant correlation was found between anti-GP2 IgA and CD-specific antibodies with a Spearman's coefficient of rank correlation (rho) for EmA of 0.494 and for anti-tTG IgA of 0.466 (p < 0.001, respectively) (Fig 1). In contrast to anti-GP2 IgA, ASCA IgG, which was also more prevalent and elevated in active CD patients, was neither correlated with EmA nor with anti-tTG IgA or IgG (p > 0.05 respectively).

### Behavior of GP2-IgA in the course of CD

**Fig 2:**
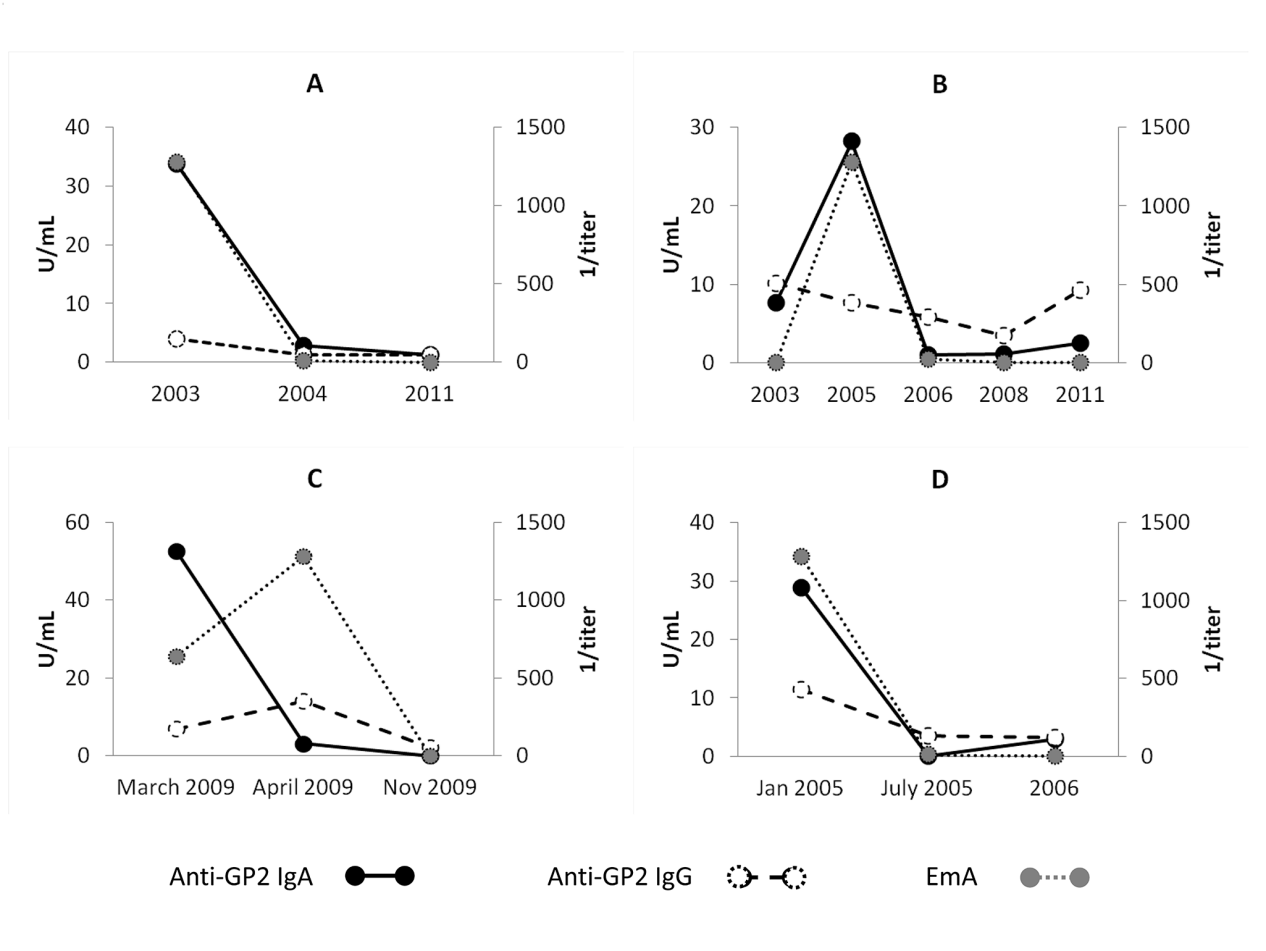
Antibody kinetics in 4 celiac disease patients. Antibody kinetics in 4 celiac disease patients (A-D) with at least two consecutive samples before and after the onset of a gluten free diet. In fact, in all patients anti- glycoprotein 2 (GP2) IgA levels were reduced to values below the cut-off. Interestingly, in one patient (patient B) with co-existing type 1 diabetes who became positive for anti-GP2 IgA in parallel with anti- tissue transglutaminase (tTG) IgA, anti-GP2 IgA turned also negative under gluten free diet.

We investigated 28 CD patients with anti-GP2 positivity before and after the onset of a GFD (S1 Fig). In all 28 patients with at least one follow-up sample, values for CD-specific antibodies (anti-tTG IgA and EmA) declined and so did anti-GP2 IgA. Examples for characteristic antibody kinetics are given in Fig 2. In fact, in all patients anti-GP2 IgA levels were reduced to values below the cut-off. Interestingly, in one patient (patient B in Fig 2) with co-existing type 1 diabetes who became positive for anti-GP2 IgA in parallel with anti-tTG IgA, anti-GP2 IgA turned also negative under GFD.

### Association of anti-GP2 with degree of mucosal damage

**Fig 3:**
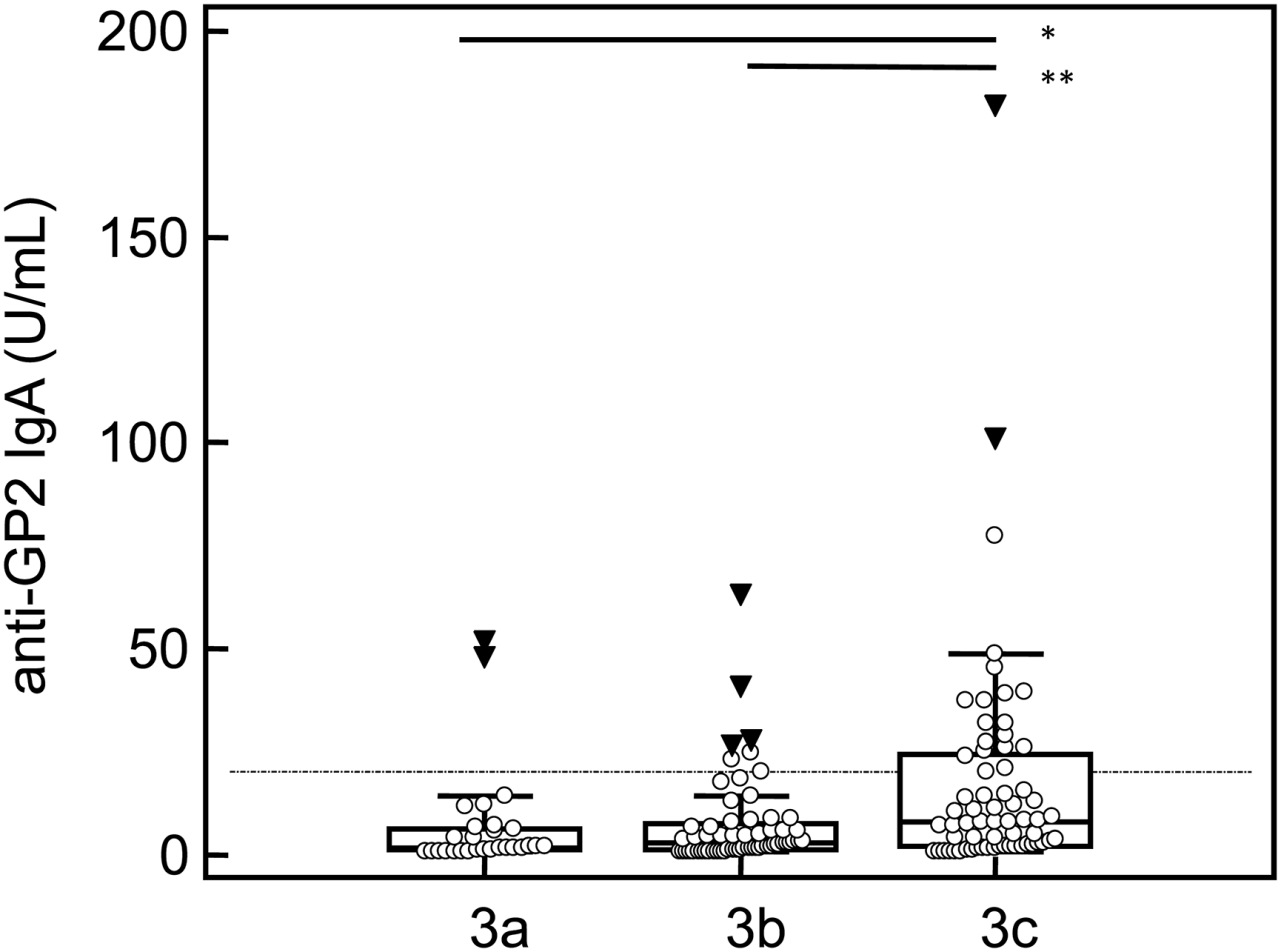
Association between anti-GP2 IgA level and degree of villous atrophy. IgA antibody levels to glycoprotein 2 (GP2) determined in 153 patients with active celiac disease are associated with the grade of villous atrophy according to Marsh classification. The dotted line indicates cut-off value of 20 U/mL for anti-GP2 IgA. Data are displayed as U/mL in Box-and-Whisker plots with far out values, defined as values that are smaller than the lower quartile minus 3 times the interquartile range, or larger than the upper quartile plus 3 times the interquartile range, displayed as solid triangles. * p = 0.001, ** p = 0.002.

Investigating 153 patients with active CD and available histology, we found a significant association between the anti-GP2 IgA level and grade of villous atrophy according to Marsh classification (Kruskal-Wallis test, p < 0.001) (Fig 3). In particular, anti-GP2 IgA of CD patients with Marsh classification 3c demonstrated a significantly higher median of 7.9 U/mL (IQR: 2.2–24.5) than those with Marsh 3a (1.8 U/mL, IQR: 1.1–6.5, p = 0.001) and 3b (3.2 U/mL, IQR: 1.4–7.4). Both CD-specific autoantibodies also demonstrated a significant association with grade of villous atrophy (p < 0.001, respectively). Neither anti-GP2 IgG nor ASCA IgA or IgG demonstrated such association (p > 0.05, respectively).

No association of anti-GP2 IgA or IgG positivity or antibody level with gender, age at diagnosis, positive family history for CD, occurrence of type 1 diabetes, and body mass index was found in CD patients. In two patients with coexisting inflammatory bowel disease (one with Crohn's disease and one with ulcerative colitis) anti-GP2 IgA and IgG were negative.

### Confirmation of autoantibody reactivity

**Fig 4:**
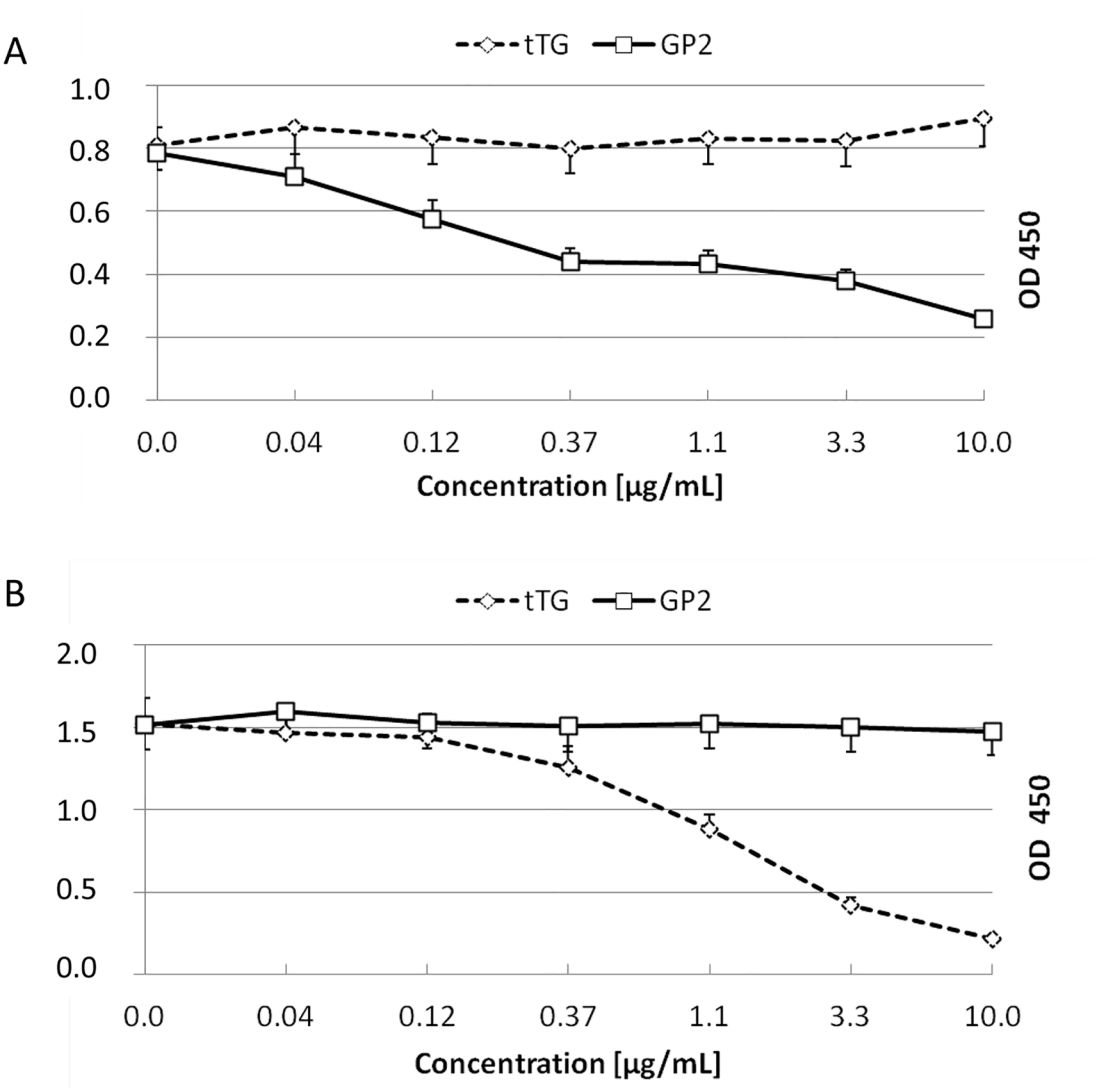
Demonstration of autoantibody specificity by inhibition in ELISA. A: Inhibition of serum IgA reactivity to glycoprotein 2 (GP2). A serum with high anti-GP2 IgA level at a dilution giving an optical density (OD) of 0.8 was pre-incubated with recombinant GP2 (Isoform alpha) and tissue transglutaminase (TG) at decreasing concentrations from 0–10 μg/mL. B: Inhibition of serum IgA reactivity to tTG. A serum with high anti-tTG IgA level at a dilution giving an optical density (OD) of 1.5 was pre-incubated with recombinant GP2 (Isoform alpha) and tTG at decreasing concentrations from 0–10 μg/mL. Data are presented as means of triplicate measurements with the corresponding standard deviation.

To confirm the serum IgA reactivity to tTG and GP2 and to exclude cross-reactivity between both autoantigenic targets, dilutions of autoantibody-positive sera were incubated with decreasing concentrations from 10.0 to 0.0 μg/mL of tTG and GP2. Subsequently, the samples were run in the anti-tTG and anti-GP2 ELISA. Incubation of anti-GP2 IgA-positive sera with recombinant tTG and anti-tTG IgA positive sera with recombinant GP2 (Isotype 1) did not decrease the respective antibody titers (Fig 4). In contrast, recombinant tTG and GP2 inhibited the binding of anti-tTG and anti-GP2, respectively, in the corresponding ELISA. This indicates that there is no cross-reactivity between anti-GP2 IgA and anti-tTG IgA.

## Discussion

In the present study we found elevated anti-GP2 in 20.1% of patients with active CD. In particular, IgA to GP2 (19.5%) was significantly more prevalent than IgG (4.5%). Anti-GP2 IgA levels correlated significantly with EmA and anti-tTG IgA levels (Fig 1). Similar to these CD-specific antibodies, we demonstrated the disappearance of anti-GP2 positivity in patients with CD in the follow-up under GFD (Fig 2 and S1 Fig). Anti-GP2 IgA seems to be associated with disease activity in a distinct subgroup of patients with CD. The observed loss of tolerance to GP2 in CD is transient and disappears under GFD. Likewise, Gross et al. demonstrated in a recent study positivity for anti-GP2 IgA in 29% of adult patients with active CD and a decrease under gluten-free diet [21]. In contrast, they found increased levels of anti-GP2 IgA in patients with refractory CD, suggesting anti-GP2 as a marker for this condition.

CD and inflammatory bowel diseases (Crohn's disease and ulcerative colitis) are the most prevalent immune mediated diseases of the gastrointestinal tract [26, 27]. CD and Crohn's disease share common genetic risk variants detected in genome-wide association studies [28, 29]. Co-occurrence of CD and Crohn's disease was described in case reports und case series. In a larger study, Crohn's disease was detected in 4% of patients with CD [30]. Pancreatic autoantibodies and recently anti-GP2 antibodies were considered specific for Crohn's disease with a sensitivity ranging from 20 to 40% [1, 31]. Thus, the occurrence of anti-GP2 antibodies in 20% of patients with active CD questions the specificity of these antibodies [19, 20]. The other main Crohn's disease-specific antibody ASCA which shows a prevalence of about 50 to 80% in patients with Crohn's disease was also found in 14% of patients with CD but only in 1.5% of healthy controls [25]. Data for the recently reported antibodies to Crohn’s-disease peptide are still lacking for CD [32]. In another study, ASCA positivity was found even in up to 49% of CD cases at the time of diagnosis [33]. Intriguingly, ASCA serum levels correlated with the degree of villous atrophy and, remarkably, decreased under GFD [34]. In contrast, we did not determine a significant reduction of ASCA in CD patients under GFD in this study. However, we found a significant reduction for anti-GP2 IgA. For the first time, we could establish a significant correlation of anti-GP2 IgA with the degree of mucosal damage in patients with CD. Furthermore, anti-GP2 IgA was significantly correlated with CD-specific autoantibodies which corroborate previous findings [23].

Thus, disappearance of anti-GP2 in CD after elimination of dietary gluten supports the assumption that anti-GP2 negativity indicates immunological remission in CD as demonstrated for CD-specific autoantibodies. In contrast, the association of anti-GP2 with disease activity in Crohn’s disease is yet controversial [12, 15, 18, 35, 36]. Pathogenesis of CD and inflammatory bowel diseases is only partly understood to date, but an abnormal intestinal barrier function leading to a leaky gut appears to be crucial in both clinical conditions. Anti-GP2 antibody and ASCA positivity may reflect this increased permeability of the intestinal mucosa. A recent study found significantly increased positivity for anti-tTG and anti-gliadin antibodies in patients with inflammatory bowel diseases compared to controls, even if CD could not be confirmed by histology in any of the patients [37].

Additionally to the exocrine pancreas, GP2 was also identified in different cells of the innate and acquired immune systems, especially in microfold cells (M cells) of the follicle-associated epithelium in the intestine [6, 38]. It can be found in the inflamed tissue of patients with Crohn’s disease and Crohn’s disease-like chronic pouchitis [39, 40]. Being one of the M cell-specific molecules in humans and mice, GP2 mediates the uptake and transcytosis of distinct bacteria by M-cells [5]. GP2 is also expressed in lamina propria T-cells and in distinct dendritic cells [41]. Bacteria opsonized by soluble GP2 might be taken up by dendritic cells [10, 41]. Since GP2 is involved in antigen presentation and immunoregulation, GP2 itself may be involved in the pathogenesis of CD. A hallmark in the pathophysiology of CD is the tolerance break to gliadin and related prolamines. M cell-mediated antigen uptake into gut-associated lymphoid tissue may facilitate induction of oral tolerance to soluble antigens [42].

Active CD is associated with impaired exocrine pancreatic function [43]. The pathophysiology of exocrine pancreatic insufficiency in CD involves a number of factors. Decreased production of cholecystokinin leads to impaired release of digestive enzymes and probably GP2 in the pancreatic juice. However, absence of GP2 in a mouse model does not affect pancreatic secretion [44]. Therefore, it is likely that also GP2 in pancreatic juice has an immunological function [45]. Remarkably, pancreatic GP2 was found to be decreased on the surface of bacteria in Crohn's disease patients compared to healthy controls [46]. This may aggravate mucosal inflammation by increased adhesion and may facilitate invasion of bacteria. Another possible pathophysiological role of anti-GP2 IgA may be the binding to and neutralizing of GP2 itself. Consequently, this would diminish the anti-inflammatory effect of GP2, which is mediated by regulatory T cells [10]. However, we did not find autoantibodies against exocrine pancreas by indirect immunofluorescence in anti-GP2 positive patients or controls. It is, thus, likely that target of these antibodies is not GP2 of the exocrine pancreas. In contrast to Crohn's disease, where anti-GP2 antibody belongs predominantly to the IgG isotype, the predominant isotype thereof is IgA.

Further studies are needed to elucidate the role of GP2 and anti-GP2 antibody in inflammatory diseases of the gastrointestinal tract and to decipher if loss of tolerance to GP2 contributes to or is brought about by intestinal inflammation.

In conclusion, this study showed that in patients with anti-GP2 positivity, especially of IgA isotype, not only Crohn's disease but also CD should be considered as differential diagnosis. In CD patients with anti-GP2 antibody positivity, this marker can be used as indicator for intestinal inflammation and for follow-up. CD should be differentiated from Crohn’s disease by parallel testing of CD-specific EmA or anti-tTG.

## Supporting Information

S1 Fig Antibody kinetics in 28 celiac disease patients under gluten-free diet. Antibody kinetics in 28 celiac disease patients with at least one sample before and one after the onset of a gluten free diet. IgA and IgG antibodies to glycoprotein 2 (GP2) IgA (red), anti-tissue transglutaminase (TG) IgA (green) and endomysial IgA antibodies (EmA) (blue) were determined by enzyme-linked immunosorbent assay and indirect immunofluorescence, respectively. The first point indicates the date of diagnosis of celiac disease and also the starting point for gluten-free diet (with exception of patient 5). In patient 5 type 1 diabetes was diagnosed in May 2003 and celiac disease in January 2005. Therefore he was first negative and became positive for EmA, anti-tTG IgA and anti-GP2 IgA. All three antibodies turned also negative under GFD. In all 28 patients anti-GP2 IgA were reduced to values below the cut-off under gluten-free diet. (Note that patients A-D in Fig 2 correspond to patients 3, 5, 9 and 25 respectively in S1 Fig. Antibody kinetics in 28 celiac disease patients under gluten-free diet).
(TIFF)

S1 Table Clinical and antibody data of patients and controls. Format is xls in 19 columns and 388 lines. Column 1: ID = identification number of participant; Column 2: status code 1 = celiac, 2 = celiac under GFD, 0 = control (Group); Column 3: age in years at diagnosis/ blood test; Column 4: titer of endomysial autoantibodies (EmA-IgA); Column 5: titers of autoantibodies to tissue transglutaminase (anti-tTG) IgA; Column 6: titers of autoantibodies to tissue transglutaminase (anti-tTG) IgG; Column 7: titers of anti-glycoprotein 2 (anti-GP2) IgA; Column 8: titers of anti-glycoprotein 2 (anti-GP2) IgG; Column 9: titers of anti-Saccharomyces cerevisiae antibodies (ASCA) IgA; Column 10: titers of anti-Saccharomyces cerevisiae antibodies (ASCA) IgG; Column 11: gender code (1 = male, 2 = female); Column 12: gender: female (f) or male (m); Column 13: Marsh Code (3 = Marsh IIIa, 4 = MarshIIIb, 5 = Marsh = IIIc); Column 14: histology at diagnosis of celiac disease (Marsh classification); Column 15: diabetes mellitus type 1 (0 = no, 1 = yes); Column 16: celiac in first degree family member? (0 = no, 1 = yes); Column 17: BMI in kilogram/m^2^ at diagnosis; Column 18: BMI percentiles; Column 19: BMI as standard deviation score (SDS).
(XLS)
