# Supplementary figures and images for: Loss and Gain of Tolerance to Pancreatic Glycoprotein 2 in Celiac Disease
Source: PLoS One. 2015 Jun 5;10(6):e0128104. doi: 10.1371/journal.pone.0128104 (PMC4457647; doi:10.1371/journal.pone.0128104)

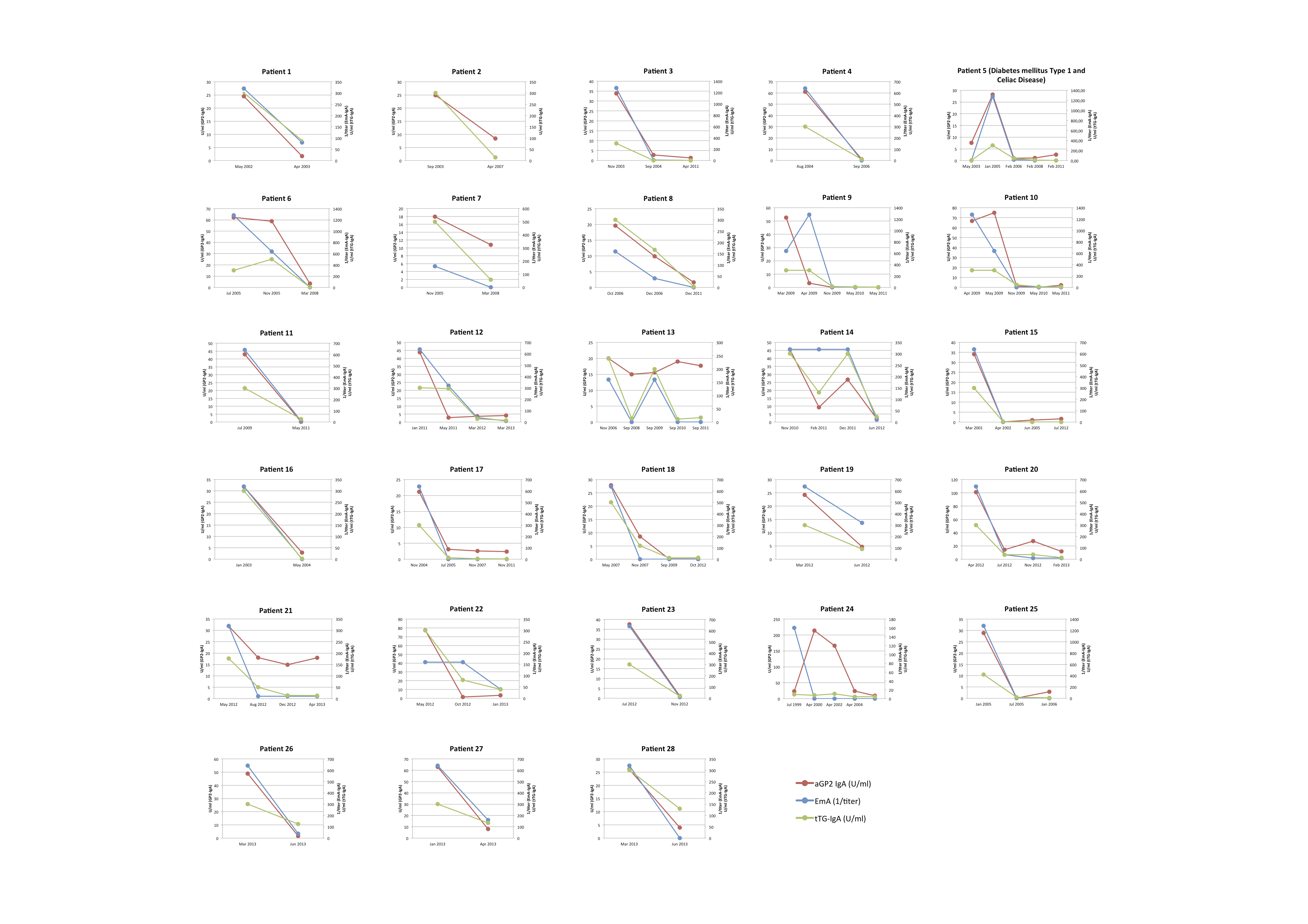

Supplement: S1 Fig — Antibody kinetics in 28 celiac disease patients with at least one sample before and one after the onset of a gluten free diet. IgA and IgG antibodies to glycoprotein 2 (GP2) IgA (red), anti-tissue transglutaminase (TG) IgA (green) and endomysial IgA antibodies (EmA) (blue) were determined by enzyme-linked immunosorbent assay and indirect immunofluorescence, respectively. The first point indicates the date of diagnosis of celiac disease and also the starting point for gluten-free diet (with exception of patient 5). In patient 5 type 1 diabetes was diagnosed in May 2003 and celiac disease in January 2005. Therefore he was first negative and became positive for EmA, anti-tTG IgA and anti-GP2 IgA. All three antibodies turned also negative under GFD. In all 28 patients anti-GP2 IgA were reduced to values below the cut-off under gluten-free diet. (Note that patients A-D in Fig 2 correspond to patients 3, 5, 9 and 25 respectively in S1 Fig. Antibody kinetics in 28 celiac disease patients under gluten-free diet). (TIFF) [file pone.0128104.s001.tiff]
